# The Analysis and Risk Assessment of Organic Pollutants in Food Products

**DOI:** 10.3390/foods14183168

**Published:** 2025-09-11

**Authors:** Serenella Seccia, Irene Dini

**Affiliations:** Department of Pharmacy, University of Naples Federico II, Via Montesano 49, 80131 Napoli, Italy; seccia@unina.it

Unsustainable development practices have caused significant harm to the world’s ecosystems. Every day, our water bodies are contaminated with a variety of organic pollutants, including pesticides, antibiotics, dyes, disinfectants, food additives, and nitrophenols, which are often discharged without adequate treatment [1]. These non-biodegradable substances persist in the environment, accumulating in living organisms and infiltrating the food chain [2]. The consequences are alarming: ecosystems are becoming increasingly destabilized, and human health is at significant risk. This Special Issue presents a carefully curated collection of innovative research articles and comprehensive reviews that aim to identify harmful substances in animal-derived foods, offering new insights into food safety monitoring. It features cutting-edge methods for detecting pesticide residues in wheat [3] and dairy products such as yogurt [4]. Pesticides are used in agriculture to control weeds and pests, but they can interfere with the human reproductive [5,6,7,8] and immune systems [7], leading to certain metabolic disorders, particularly with long-term exposure through contaminated food and water [8,9]. The issue also explores the widespread use of antibiotics, which, when consumed excessively or without regulation, can trigger hypersensitivity, allergies, gut microbiome disturbances, aplastic anemia, and a rise in antibiotic-resistant bacteria.

Antibiotic residues not only affect human health but also pose significant economic challenges for the dairy sector. These substances can interfere with crop growth, which is crucial for dairy production [10,11,12]. To address this, the issue introduces a novel analytical method for measuring antibiotic levels in milk [13], offering a promising tool for industry-wide safety checks.

Furthermore, the unchecked use of antibiotics in agriculture, livestock, and industrial wastewater has accelerated the emergence of antibiotic-resistant microbes [14,15]. This issue examines their presence in food to enhance surveillance systems and develop practical strategies for protecting public health.

Ultimately, this Special Issue aims to enhance food safety standards by advocating for novel analytical techniques to detect pesticides, antibiotics, and antibiotic-resistant organisms, thereby fostering healthier and more transparent food systems for future generations. To bolster these initiatives, the authors emphasize the pressing need for advanced toxicological research, a critical step in ensuring maximum consumer protection.

## Data Availability

No new data were created or analyzed in this study. Data sharing is not applicable to this article.
